# Mineral-Enriched Postbiotics: A New Perspective for Microbial Therapy to Prevent and Treat Gut Dysbiosis

**DOI:** 10.3390/biomedicines10102392

**Published:** 2022-09-25

**Authors:** Laura-Dorina Dinu, Ionela Avram, Diana-Roxana Pelinescu, Emanuel Vamanu

**Affiliations:** 1 Faculty of Biotechnology, University of Agricultural Sciences and Veterinary Medicine, 011464 Bucharest, Romania; 2 Faculty of Biology, University of Bucharest, 030018 Bucharest, Romania

**Keywords:** mineral-enriched postbiotics, microbiota modulation, gut dysbiosis, mineral bioavailability, microbial therapy

## Abstract

Postbiotics are non-viable probiotic preparations that confer a health benefit on the host. In the last years, scientific literature has proved that postbiotics have health-promoting features and technological advantages compared to probiotics, augmenting their full potential application in the food and pharmaceutical industries. The current work comprehensively summarizes the benefits and potential applications of postbiotics and essential mineral-enriched biomass and proposes a new strategy for microbial therapy—mineral-enriched postbiotics. We hypothesize and critically review the relationship between micronutrients (calcium, magnesium, iron, zinc, selenium) and postbiotics with gut microbiota, which has been barely explored yet, and how the new approach could be involved in the gut microbiome modulation to prevent and treat gut dysbiosis. Additionally, the bioactive molecules and minerals from postbiotics could influence the host mineral status, directly or through gut microbiota, which increases the mineral bioavailability. The review increases our understanding of the health improvements of mineral-enriched postbiotics, including antioxidant functions, highlighting their perspective on microbial therapy to prevent and threaten gut-related diseases.

## 1. Introduction

Human-associated microorganisms—approximately 10^14^ species—including their genes and metabolites, are known as microbiota, and their configuration begins from the perinatal period [1,2]. In the case of intestinal microbiota, the interdependent relationship with the human gastrointestinal tract is established during the first 3 years of life, but several important factors (e.g., genetics, diet, stress) can influence microbiota composition and, subsequently, their powerful symbiosis with the human body [2]. The imbalance in the gut microbiota, known as dysbiosis, can lead to serious consequences: intestinal and extra-intestinal disorders such as allergic/autoimmune diseases, cardiovascular or neurological disorders, behavioral diseases, or cancer [1,2,3]. Therefore, prevention and therapeutic strategies that maintain a healthy gut environment and human well-being have become trendy.

The “probiotic paradox” is based on the observation that both live and dead probiotic cells can produce a biological response, suggesting that the consumption of non-viable microbial cells confers some health benefits for consumers [3]. These ideas have directed microbiota research towards the postbiotic concept. Another direction explored the contribution of gut microbiota in the biosynthesis, uptake, absorption, and bioavailability of micronutrients. Therefore, the present paper aims to present an overview of the developments in the area of postbiotics related to advances in research of micronutrient-enriched biomass, and gaps in our knowledge are highlighted while proposing a new strategy for microbial therapy—mineral-enriched postbiotics. The possible beneficial effects of these products on the host, directly or through the gut microbiota, are described and discussed based on the existing evidence about relationships between postbiotics and minerals with the gut microbiome, and further directions are depicted.

We performed a literature search using Boolean operators of original and review articles in English as a research method. To identify studies addressing this topic, the search was conducted with the following databases: Google Scholar, Scopus, and PubMed. This manuscript’s search terms to find suitable papers for this manuscript (especially between 2015 and 2020) were: novel targets in postbiotics, micronutrient-enriched biomass, gut microbiota modulation, mineral bioavailability, biotherapy, molecular pathway, and clinical evaluation. No particular limitations were taken into consideration.

## 2. The Health Benefit of Postbiotics and Their Applications in Microbial Therapy to Prevent and Treat Gut Dysbiosis

### 2.1. Postbiotic Concept

Although the first mention of the term probiotics was in 1953; the panel experts from Food and Agricultural Organization (FAO) and World Health Organization (WHO) defined probiotics in 2001 with some minor amendments in 2013 as “live microorganisms that, when administered in adequate amounts, confer a health benefit on the host” [4]. In the last 20 years, studies on probiotic microorganisms have grown, and with them, consumer interest has developed [3,5]. According to research market analysis, the global probiotics market in 2020 was valued at $34.1 billion and is projected to grow to $73.9 billion by 2030 [5]. This is due, in part, to the general trend to improve health and prevent diseases based on consuming either functional foods or dietary supplements, and, on the other hand, due to numerous experimental data that have shown the beneficial effect of microorganisms.

Studies regarding mechanisms involved in the probiotic effect revealed that it is partially due to cell parts or metabolites released by probiotic microorganisms. Therefore, in 2013, after 10 years of probiotic definitions by FAO and WHO, a new term was introduced for postbiotic: “any factor resulting from the metabolic activity of a probiotic or any released molecule capable of conferring beneficial effects to the host in a direct or indirect way” [6]. The term was used in the scientific literature and on commercial products, but at present, there are various scientific opinions on the definition of postbiotics and no consensus yet [3,7,8]. The International Scientific Association for Probiotics and Prebiotics (ISAPP), in 2019, defined postbiotics as the “preparation of inanimate microorganisms and/or their components that confers a health benefit on the host” that should contain inactivated microbial cells or cell components with or without metabolites [9]. Based on this definition, postbiotics are not necessarily derived from probiotic microorganisms, they can be obtained as a result of the inactivation of any living microorganism, and not all probiotics could be used as postbiotics after killing them [10]. The term postbiotic used in this paper is in accordance with this generally accepted definition.

Other authors consider this definition inappropriate due to some unclear issues and overlap with the term paraprobiotics [6,7]. In the same year (2021), Siciliano and collaborators proposed the following definitions:

- Postbiotic—“compounds derived from microbial metabolism synthesized by cells or produced in the matrix by enzymatic action”;
- Paraprobiotic—“inactivated microbial cells (non-viable), specifically, cells as a whole, including both structural components and synthesized or excreted metabolites that confer a health benefit to the consumer” [7].

### 2.2. Postbiotic Advantages and Beneficial Effects on Human and Animal Health

In both cases, either there are metabolites or inactivated cells/components of cells (most of them from probiotic strains), and many in vitro or in vivo studies have shown the beneficial effect of these products. The beneficial effects of postbiotics in terms of improving health or preventing disease in humans and animals are shown in Table 1. Moreover, in recent years, postbiotics have been considered a novel approach for cancer treatment as adjuvant therapy and in the treatment of gut-related diseases and infections, such as tuberculosis [11,12,13,14].

There are some advantages regarding the utilization of postbiotic products instead of living probiotic microbial strains, which include: the considerably reduced risks of infection in the case of immunocompromised consumers; low risk of bacterial translocation and transfer of antibiotic resistance genes or genes involved in pathogenicity and virulence; increased stability for a longer time period; clear chemical structure and easier possibility of dosing; less interference with food characteristics (organoleptic characteristics, texture, s.o); easier to standardize, transport, and store [12,33]. Antibiotic therapy is often combined with the administration of probiotics to stabilize the gut microbiota, but there is a risk that the antibiotic will kill the bacterial strain, so the beneficial effect of the probiotics will be reversed. By using postbiotics, this risk of losing bioactivity when administered in combination with antimicrobials is eliminated. Bacterial strains have multiple gene transfer mechanisms, so probiotics could become vectors for the transfer of antibiotic-resistance genes or other genes associated with virulence factors. Another risk is that factors such as low immunity or physiological and structural changes in the host epithelial barrier may allow the entry of commensal microorganisms into the blood circulation, leading to the development of systemic infections. By 2021, more than 100 cases of patients who have developed infections caused by strains generally recognized as safe (GRAS), such as *Saccharomyces*, *Lactobacillus, Bifidobacterium*, *Bacillus*, *Pediococcus*, *Lactococcus*, and *Escherichia* sp. was reported [7].

Beyond the advantages mentioned above, the effect of postbiotics depends on many factors, which involve: the strain and substrates used for fermentation, the applied method for obtaining the final products, and the matrix in which it is incorporated (in the case of nutraceutical foods) [34]. Different technologies were applied for preparing either inactive microbial cells or cell components: heat, high pressure, oxygen exposure, chemical treatments (e.g., formalin, enzymes), gamma or ultraviolet irradiation, and sonication [9,12,33]. Although the definition of postbiotics is new, researchers have long tried to elucidate the mechanisms by which living microorganisms positively influence the health of the human host by trying to identify the compounds that underlie these effects. The mechanisms involved in beneficial effects are not elucidated, but progress is being made in this direction due to its high applicability potential in the food and pharmaceutical industry [34,35,36,37,38,39,40].

On the market, there are commercial postbiotics in functional food products or as food supplements, which intend to support the immune and digestive systems. There are studies that aim to find solutions for improving the beneficial effects of postbiotics and paraprobiotics by making complex combinations with other compounds, such as prebiotics [28].

## 3. The Health Benefit of Mineral-Enriched Biomass and Their Applications in Microbial Therapy to Maintain Eubiosis and Improve Mineral Bioavailability

Micronutrients are vitamins and minerals needed by the body in very small amounts. However, their impacts on the body’s health are critical, and deficiency in any of them can cause severe and even life-threatening conditions. Because humans cannot synthesize all essential micronutrients, they need to be acquired exogenously from diet or oral supplements or produced by intestinal microbiota, such as vitamins K and B group [41]. They perform a range of functions, including enabling the body to produce enzymes, hormones, and other substances needed for normal growth and development. Micronutrient deficiencies can cause visible and dangerous health conditions, but they can also lead to less clinically notable reductions in energy level, mental clarity, and overall capacity. Many of these deficiencies are preventable through nutrition education and consumption of a healthy diet containing diverse foods, as well as food fortification and supplementation, where needed.

### 3.1. The Impact of Micronutrients on Human Health

Micronutrients ensure the preservation of homeostasis with a key role in the response that the human body can have in case of interactions with the external environment or reduce the disease burden. Certain micronutrients (vitamins and/or carotenoid compounds) are strictly dependent on the concentration present in the body to be considered valid. Often, their low concentration makes their influence negligible in the proper functioning of physiological functions [42]. Usually, a significant intake of micronutrients is due to the consumption of foods enriched with vitamins or minerals (such as rice or flour) [43,44]). Supplementation of various foods with micronutrients in powder form has been a common way of administering vitamins or minerals to target groups of the population, especially in products suitable for the prevention or treatment of anemia [45]. A study based on the Multiple Source Method in the elderly showed that the intake of folic acid, calcium, vitamin B6, and vitamin B2 was insufficient. In contrast, sodium intake was increased in the above-mentioned age group [46]. The same study had several conclusions valid throughout Europe, in that socioeconomic status was directly correlated with the amount of micronutrients consumed by food. High consumption of vitamins A, B2, and C was directly associated with the socioeconomic status of the target group. What matters in the case of target groups with age-appropriate nutritional requirements and specific diseases is nutrition education [46]. This depends on the degree of education, economic development of the country, and awareness that increasing food consumption does not mean a good state of health. To have a correct nutritional yield, even at an advanced age, a balance must be taken into account between what we consume, the general state of health, and the possibility of access to products with an appropriate degree of quality. Overall, social status, lifestyle, and eating habits influence the proper functioning of the body and the response we have in the case of interaction with a pathogen [47]. Not only vitamins are essential for the proper functioning of the immune response (e.g., vitamin C). Folic acid, calcium, magnesium, zinc, iron, copper, and selenium act independently and in direct relation to each other to support this essential function and maintain homeostasis. Moreover, the level of each micronutrient is a critical detail. The additional intake of these micronutrients currently solves the response to exogenous factors, and the way they are administered determines their bioavailability. Maintaining homeostasis has become an issue in recent years because micronutrients and other bioactive compounds play a role in stimulating the cells of the immune system, which plays an essential role in resistance to SARS-CoV-2, for example. Controlled administration is part of the current strategy for maintaining the functioning of the immune system [48]. Minerals (e.g., selenium and/or zinc) and bioactive extracts from curcumin, echinacea, and propolis have been partially recognized as having possible actions in combating and reducing the effects of SARS-CoV-2 infection [49]. These data mentioned an action on the angiotensin-converting enzyme, inhibition of papain-like protease or chymotrypsin-like protease, and action against RNA-dependent RNA polymerase involved in the coronavirus replication cycle. The effects are also recognized against some dysfunctions associated with a viral infection, for example, severe acute respiratory syndrome. The mode of action of these micronutrients is manifested through the anti-inflammatory, antioxidant, antiviral, or immunomodulatory properties. Starting from reducing inflammatory processes, micronutrients and bioactive substances are an affordable option that effectively supports the fight against COVID-19 [49].

### 3.2. Mineral-Enriched Biomass Obtaining and Advantages

A new perspective, less considered, is the use of essential minerals to enrich the biomass of yeast and/or bacteria. Such a process would be helpful because it leads to the development of innovative new generation products, such as mineral-enriched prebiotics and postbiotics. Thus, it is possible to supply the human body with a large quantity of minerals (such as calcium, magnesium, iron, zinc, and selenium) with high bioavailability for the organism, in addition to the complex and beneficial components from the pre/postbiotic composition. Fermentation incorporation is an essential step in obtaining these products. Moreover, the natural assimilation of minerals by microbes improves the value of biomass as microbes are naturally adsorbent for minerals and increase micronutrient bioavailability, while the process is eco-friendly and cost-effective [41,50].

An important criterion for selecting industrial microbial strains for mineral-enriched biomass production is their stress tolerance, including the stress produced by the elevated concentration of one or two mineral ions and various stresses from the intestinal tract. A study showed that *Saccharomyces cerevisiae* had a lower resistance than *Kluyveromyces marxianus* to the presence of free radical species, also encountered in the gut. This detail makes the fermentation yield optimal at high temperatures, which has led to an advantage that can be used in innovative fermentation processes [51]. Some lactic acid bacteria (LAB) have been reported to be able to resist high concentrations of inorganic selenium (200 mg/L) and reach the maximum bioaccumulation potential in a medium with 150 mg/L of Se [52]. In this condition, two *Lactobacillus paracasei* strains (ML13 and CH135) were able to bioaccumulate approximately 40 mg Se/g biomass. Optimization studies to produce Se-enriched *Saccharomyces boulardii* CCT 4308 probiotic biomass in a batch system noted that the best Se accumulation was observed at 100 μg/mL while the strain can tolerate 400 μg/mL, and a good strategy to avoid a metal toxic effect is the addition of selenium at the end of the logarithmic phase [53]. In this case, the best biomass production (14.52 g/L) was obtained after 12 h cultivation, and cells accumulated 3.2 mg Se/g biomass, a result comparable with the selenium bioaccumulation by some commercial probiotics. Natural mediums were used to grow Se-enriched yeasts, sugar molasses—a complex carbon source with small amounts of proteins, vitamins, and nitrogen compounds in the case of *S. boulardii*—and juices from germinated brown rice, beewort, and soybean sprouts in a ratio of 4:4:2 for *S. cerevisiae*, where different concentrations of sodium selenite were added [53,54]. *S. cerevisiae* grew with lower amounts of inorganic selenium (15 μg/mL Na_2_SeO_3_) but produced 8.5 g/L biomass, and total Se achieved 3.53 mg/L. The carbon sources clearly influence the bioaccumulation process in *S. cerevisiae*; when in a medium with beet and sugarcane molasses, a strain yielded 3.77 mg/g intracellular selenium, while in synthetic and industrial media, reported 2.72 mg/g and 2.46 mg/g Se accumulation, respectively [54,55]. Another work proved that *S. cerevisiae* strains are able to adapt to growing in the presence of four times higher concentrations of minerals such as selenium, but macromorphological changes in the yeast colonies and growth reduction were observed during the adaptation process [56]. Interestingly, an adapted Zn-enriched *Lactobacillus plantarum* strain showed stronger tolerance to stress produced by acid, bile salts, and hydrogen peroxide, while antioxidant properties increased significantly [57]. In this case, electron microscopy analysis showed that the ultrastructure of zinc-enriched strain changes, as well as the metabolites pattern, compared to the non-adapted control strain. Different from selenium accumulation, yeasts are able to accumulate a higher quantity of inorganic zinc than bacteria, as Zn is an essential element for *S. cerevisiae* growth. However, in the presence of excessive ZnSO_4_, this element had inhibitory effects [58]. The total Zn accumulation gradually gets bigger until there is 30 mg/L of zinc sulfate in the medium, while the biomass increased by 24-fold. Similar results were reported with an *S. cerevisiae* strain isolated from industrial sewage that, in optimal conditions of 25 μg/mL of zinc, at pH 6 and after 24 h of incubation, showed the maximum growth and Zn uptake, while the protein content of the biomass was above 50% (*w*/*w*) [59]. Furthermore, cultivation conditions are important in the case of zinc absorption, as the pH-dependency of Zn uptake has been reported [59,60].

In the last years, commercial preparations with selenium, zinc, multi-vitamins, and probiotic strains have been available.

### 3.3. Minerals—Gut Microbiome Relationship and the Beneficial Effects of Mineral-Enriched Biomass on Human and Animal Health

In healthy humans, minerals that are not absorbed in the small intestine get to the colon and become available for the commensal gut microbiota. The main effect of these minerals is to act/modulate the microbiota in the gut, both in terms of the microbial fingerprint and especially through the metabolic processes that can directly affect human health [61]. Some of the secreted microbes’ metabolites will contribute to the host’s mineral status improving the uptake, absorption, and bioavailability of minerals [41]. The dynamic relationship between the modulation process of the microbiota and the bioavailability of micronutrients is a process of interest in microbial therapy, but also for characterization of functional foods and the realization of personalized feeding schemes [61,62].

Recent works in humans and different model organisms demonstrated that the mineral–gut microbiome relationship is bidirectional. On the one hand, there is a symbiotic relationship between intestinal microbiota and the host that influence eubiosis and mineral status of the host. Bacteria can produce enzymes that help release minerals from foods, such as phytase enzymes that induce plant phytic acid hydrolysis releasing minerals, such as calcium, phosphate, and magnesium [63]. Microbes in the gut use minerals for their growth and functioning, avoiding health impairment associated with dysbiosis and pathobiont overgrowth [41]. For instance, phosphorous supplementation increased the microbial diversity and levels of short-chain fatty acids (SCFA), microbial metabolites produced from polysaccharide fermentation in the colon [64]. SCFA plays an important role in neuro-immunoendocrine regulation in humans. Zinc deficiency decreases the biodiversity of gut microflora, negatively alters their function and gut-brain signaling and increases inflammatory markers in the host’s blood system [65]. Interestingly, clinical trials with iron supplementation negatively modulated gut microbiota, increasing the proportion of enteropathogen *E. coli* while LAB (bifidobacteria and lactobacilli) relative abundance decreased [66]. The presence of various commensal bacteria in the gut and some end-products of microbial fermentations, such as SCFA, seem to favor mineral absorption. A study showed that an increase in the presence of SCFA-producing species, such as *Bifidobacterium* sp. or *Lactobacillus* sp., was correlated with the increase in the calcium absorption that influences the bone density and strength in humans and animals [67]. Furthermore, it has been shown that iron absorption is influenced by SCFA and bacterial siderophores facilitate the host uptake of iron [68,69]. Selenium supplementation induces changes in the composition of gut microflora that protect against intestinal dysfunction; at the same time, intestinal microflora improves the bioavailability of selenocompounds [70]. On the other hand, in the case of mineral limitations, gut microbes and the host become competitors; for instance, in selenium-limiting conditions, intestinal bacteria can remove Se and lower the selenoprotein level in the host and eventually impair the host immune response [70].

Overall, these findings demonstrate the importance of microbiota as a feasible target for improving host mineral bioavailability. It is, therefore, not surprising that probiotic supplementation was used in the treatment of mineral deficiency. In the last 10 years, in vivo studies and small clinical trials have demonstrated the association between gut microbiota, probiotics, and host mineral status [41,70,71]. However, large clinical trials are missing, and the existing data are mostly the result of in vitro research [72]. The few available clinical trials in humans demonstrated that probiotics with *Lactobacillus* spp. increased the magnesium bioavailability after cheese and vegetable milk consumption or non-dietary iron absorption [73,74]. A synbiotic containing *Lactobacillus* and *Bifidobacterium* species raised blood zinc levels [75].

In the case of mineral-enriched probiotic biomass, few works in different animal models suggested their beneficial effects on health. Yang et al., 2021 used *Bacillus subtilis* yb-114246, a strain with probiotic effect previously isolated from chickens and enriched with Se by adding sodium selenite into the culture medium, then supplemented the broiler chick diet [76]. The Se-enriched probiotic strain colonized the distant segments of the ileum as proved by fluorescence in situ hybridization FISH and real-time PCR and improved bacterial diversity, increasing the number of species from *Actinobacteria*, especially *Lactobacillus*, *Peptococcus*, *Butyricicoccus*, and *Rominococcaceae* species, that improved immunity, and higher body weight. Furthermore, the proportion of conditioned pathogens or pathogens (*Salmonella*, *Shigella*, *Vibrio cholerare*) significantly decreased. Diet supplemented with inorganic Se showed no significant increases in the final body weight of the broiled chickens, while chicks receiving a Se-enriched probiotic diet had higher weight compared to those fed only with the *B. subtilis* probiotic strain, proving the synergistic effect of minerals and probiotics. In another study in a murine model, a diet supplemented with Se/Zn-enriched *Lactobacillus plantarum* probiotic changes the gut microbial composition in a different way compared to a diet with inorganic Se/Zn supplements [77]. Mice diet supplementation with Se/Zn-probiotic increased the relative abundance of *Adlercreutzia*, while the abundance of *Allobaculum* decreased compared to results obtained with other mice groups [77]. Both Se/Zn-enriched diets induced the increase of *Lactococcus* spp., mostly in mice fed with inorganic Se/Zn, and *Lactococcus lactis* has been proved to uptake zinc and is capable of selenium biotransformation [77,78]. Some LAB, especially *Lactobacillus* strains, have been shown to accumulate intracellularly and then transform toxic selenite into selenium amino acids, such as selenocysteine found in the active site of important selenoproteins involved in the regulation of the redox signaling in all living organisms [77]. The most important selenoenzymes are glutathione peroxidases, thioredoxin reductase, or deiodinases, which are involved in different processes, including antioxidant function [70]. Similarly, zinc is required for the activation of many enzymes with antioxidant activity and has demonstrated Zn uptake by yeasts and lactic acid bacteria [57,59]. The selenium and zinc blood levels were the highest after Se/Zn-enriched *L. plantarum* diet, which increased the antioxidant defenses against oxidative stress [77]. Interestingly, the pattern of the selenocompounds in endogenous lactic acid bacteria isolated after inorganic Se/Zn supplementation was different compared with results obtained in experiments with Se/Zn-bioaccumulated probiotics. In a canine model, 2 g of Se/Zn-enriched probiotic (*Lactobacillus* and *Candida utilis*) diet increased the amount of lactic acid bacteria (*Lactobacillus* and *Bifidobacterium*) in the feces, while numbers of *E. coli*, *Staphylococcus*, and *Enterococcus* decreased [79].

Through a randomized controlled study, it has been shown that an increase in micronutrient intake reduces the influence of dysbiosis in degenerative diseases [80]. Considering the role of the intestinal microbiota in reducing inflammatory processes and modulating the intake of macro- and micronutrients, a direct relationship was identified between the impact of eating habits (regular consumption of fat and sugar), micronutrients (vitamins C, E, and D, carotenoid compounds, zinc) and omega-3 fatty acids. This study concluded that these nutrients are essential for the intestinal microbiota, being directly related to the risk and progression of degenerative diseases, and bioavailability is a key factor. Dysbiosis is one of the main limiting factors supporting oxidative stress in the gut, identified as a contributing factor to developing various diseases and cancer [80,81].

## 4. Mineral-Enriched Postbiotics and Their Applications in Microbial Therapy to Prevent and Treat Gut Dysbiosis

Concerning current evidence, we propose that postbiotics enriched with essential minerals will combine the pharmacodynamic features of postbiotics and micronutrient-enriched biomass. The synergistic effect will increase the beneficial health effects of these products that exhibit enhanced safety and several practical/technological benefits. The mineral-enriched postbiotic action might be directly on the host cells as a result of the enhanced interaction of released molecules from disrupted cells and lysates with gut epithelial cells. Indirectly, minerals and postbiotic compounds might act through the gut microbiota, which influences the mineral absorption in the gastrointestinal tract and will increase the mineral bioavailability, which finally will contribute to maintaining host homeostasis.

**Figure 1:**
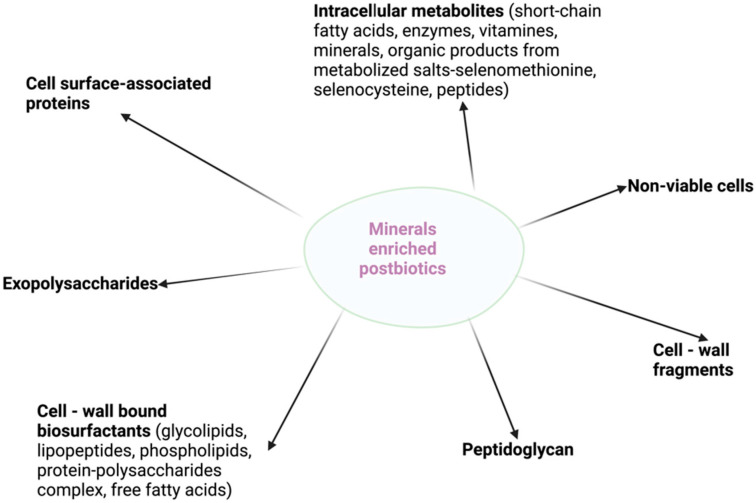
Molecules produced by mineral-enriched postbiotics.

The ability to modulate gut microbiota and produce beneficial effects for the host is a key feature of postbiotic and mineral action (Figure 1). The intestinal microbiota can use the active molecules from non-viable probiotic preparations and minerals for their growth and functioning, which in turn will influence host health. Biopolimeric cell-wall bound molecules, such as biosurfactants, exopolysaccharides synthesized and secreted by microbes during their growth, and cell surface proteins are the most important components of the cell envelope structures that play a crucial role in the molecular mechanism of postbiotics. Similarly, cell wall structural elements: peptidoglycan layer, teichoic acid, and cell-wall polysaccharides, which are present most abundantly among *Lactobacillus*, were reported to be involved in the dynamic postbiotic effect [12,82]. Moreover, a wide range of products and byproducts of alive probiotics, molecules from cell-free supernatant (bacteriocins, organic acids), and soluble factors have been found to influence gut microbiota [2]. Researchers have shown that molecules from postbiotics change the imbalanced microbiota and subsequently have positive effects on the host [7,82]. Selenium, zinc, magnesium, calcium, and iron are used by most bacteria, including the gut microflora, in metabolic processes, cellular respiration, and DNA replication. For instance, important species of bacteria that colonize the gastrointestinal tract of humans and animals (*Lactobacillus*, *Escherichia coli*, *Clostridia*, *Enterobacteria*) have genes that encode selenoproteins; thus, these bacteria metabolize and incorporate inorganic or organic selenocompounds from diet/supplements [70]. Different studies concluded that dietary Se induced changes in the composition of gut microbiota and the colonization of the gastrointestinal tract [83,84]. Moreover, recently, works have proved that selenium or selenium/zinc-enriched probiotics significantly modulate the intestinal microbiome and subsequently increase the bioavailability and absorption of these minerals in the host when compared to the inorganic forms [76,77,78]. In the case of mineral-enriched postbiotics, bioactive molecules and minerals trapped in the exopolysaccharide matrix might play a role in enhancing the biological activity of these products.

Bacterial strains related to microbiota modulation that increase mineral bioavailability are mainly LAB strains, especially *Lactobacillus* spp. and *Bifidobacterium* spp. found both in endogenous microbiota and used to manufacture probiotics and postbiotics [71]. The mineral-enriched LAB postbiotic preparations that bioaccumulate minerals as organic compounds might generate an available substance pool for the host in the large intestine, as was demonstrated for selenium-enriched probiotics [70]. On the other hand, the release of LAB microbial metabolites from pre- or postbiotics, such as SCFA, changes the intestinal ecosystem, lowering pH and making calcium more absorbable, but these molecules also facilitate the absorption of more cations (Mg, Fe^2^) [67,68,69,71]. Other metabolites, namely DAP (1,3-diaminopropane) and reuterin, antimicrobials produced by probiotic *Lactobacillus reuteri*, or metabolites such as propionate have been demonstrated to be directly involved in the iron transport in enterocytes [85]. Moreover, minerals themselves from enriched postbiotics might provide a prebiotic effect and change the composition of endogenous strains from imbalanced microbiota.

**Figure 2:**
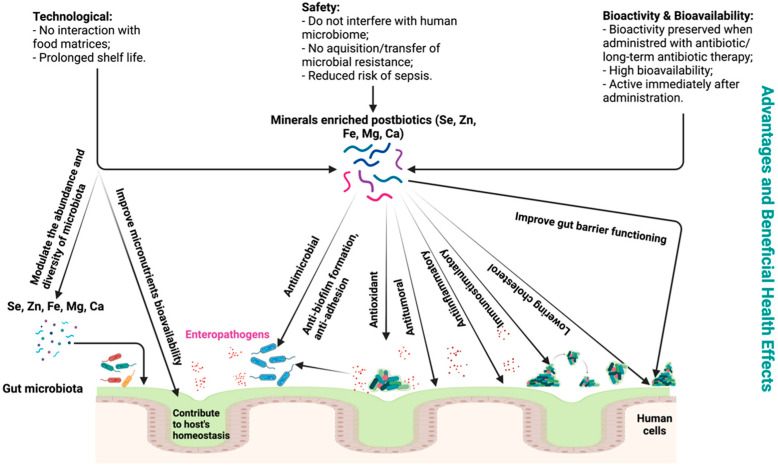
Mineral-enriched postbiotic advantages and beneficial health effects.

Both postbiotics and mineral-enriched biomass proved to have pleiotropic properties that are associated with beneficial health effects (Figure 2). Therefore, deepest studies and large clinical trials involving the biological functions and bioavailability of mineral-enriched pre- and postbiotics are highly recommended in order to confirm the findings of preclinical works.

Some postbiotics display antimicrobial and anti-adhesion activities by sealing the intestinal barrier, changing the local environment, or competing with pathogens for binding receptors [2]. Postbiotic molecules produced by *Lactobacillus sakei* EIR/CM-1 isolated from cow milk microbiota were found effective against pathogens that cause mastitis based on their antibacterial and anti-biofilm activity [18]. In the case of mineral deficiency, the gut microbiota is more susceptible to dysbiosis that promotes pathogen infection by *Salmonella typhimurium* while the host adaptative immune response is impaired [84]. On the other hand, the administration of organic selenium-enriched probiotics seems to be a promising alternative to destroying pathogenic bacteria associated with colon cancer and inflammatory bowel diseases and reducing oxidative stress [86].

Postbiotics and several minerals, such as selenium and zinc, are linked to anti-tumor, antioxidant, and anti-inflammatory properties. Supernatant compounds from *L. rhamnosus* GG and *L. paracasei* IMPC 2.1 inhibited the growth and progression of colorectal and gastric cancer in in vitro models [87]. Furthermore, various postbiotic molecules have shown anti-cancer effects by apoptosis, anti-proliferative, antimicrobial, and anti-inflammatory effects [11]. For instance, short-chain fatty acids influence the regulation of oncogenes and have a protective effect on DNA transcription but also demonstrated the potential to protect the intestinal barrier that prevents or helps treat colorectal cancer [88]. Moreover, SCFA activates molecules involved in pathways that mediate the immunity and inflammation response in murine models [89]. The intestinal microbiota directly or indirectly acts on host tumorigenesis; thus, in previous years, postbiotics have received considerable attention as cancer adjuvant therapy [90]. Different studies have indicated that postbiotics have the ability to moderate the effectiveness of cancer treatment and to reduce the side effects of conventional cancer therapies (chemotherapy and antibiotic-induced diarrhea) that help cancer patient recovery. Similar to SCFA, selenium protects DNA, and it has been suggested that Se slows the aging process by protecting DNA against reactive oxygen species [91]. Both postbiotics and minerals such as selenium and zinc exhibited antioxidant activities [92].

Postbiotics and essential minerals can modulate the adaptative and innate immune systems of animals and humans. In the case of postbiotics, they can induce similar immunological responses as probiotics by activating the same pathways or using distinct mechanisms [7].

The intestinal microbiota interferes in cholesterol metabolism through various mechanisms, including deconjugation of taurine and glycine using bile salt hydrolases, alteration of the cholesterol core by dehydroxylation, dehydrogenation, and decarboxylation reactions [93,94]. Several studies suggested that postbiotics have the ability to lower cholesterol [95].

The main feature of mineral-enriched postbiotics is the microbiota modulation that increases the bioavailability of functional compounds from postbiotics and minerals. The direct effect on the host is translated by the homeostasis maintaining, which, together with the regeneration of the microbial pattern in the colon, determines a state of equilibrium through the connections it has with the central nervous system, gut–brain axis [96]. This two-way balance has the effect of controlling degenerative processes or other chronic dysfunctions, so in vivo studies are needed to describe the mechanisms involved in controlling nerve cell function and the response of the human microbiota to stressors [97]. The plasticity of this structure is the first buffer for exogenous processes that affect neuronal integrity by stimulating the accumulation of proinflammatory compounds [98]. Control of the gut–brain axis response also leads to a normalization of hormone synthesis that profoundly affects health. The elimination of oxidative stress factors through the control of proinflammatory dysbiosis is a much more attractive prospect than the use of classic probiotic products, whose effect is limited and uncontrollable. It is also not recommended to have an uncontrolled administration of products with antioxidant effects that are not fully understood and whose biological effect is not always correlated with in vitro and in vivo data [98,99].

The advantages of the mineral-enriched postbiotics are related to their bioactivity, higher bioavailability, safety, technological aspects, and low cost. Similar to postbiotics, these products do not become part of the human microbiome, but it is a safe and effective strategy to influence the structure and composition of the existing microbiome and the host mineral status. Enhanced safety is interconnected with their inability to acquire and/or transfer antibiotic resistance/pathogenic genes and reduced risk of sepsis. Moreover, bioactivity is maintained when products are administered in combination with antimicrobial substances. Mineral-enriched postbiotics are an ideal candidate with technological advantages, including prolonged shelf life when used as supplements in microbial therapy or in the case of nutraceutical foods, where there are no interactions with the food matrix. Using stress-resistant strains, there is better availability of production processes for industrial scale-up and ease in production and storage.

## 5. Conclusions

Investigations into how microbial biotherapy can modulate the microbiome to accumulate the desired compounds will open new ways to counteract the multiple human pathologies associated with microbiota imbalance, such as nutritional disorders, cardiovascular and neurodegenerative diseases, and cancer. The proposed new strategy for biotherapy mineral-enriched postbiotics is based on the additive effects of active biomolecules and essential minerals bioaccumulated by the postbiotic strains. These have the ability to modulate microbiota and improve mineral bioavailability, suggesting various beneficial effects on the host, including antimicrobial, anti-inflammatory, anti-cancer, antioxidant, and immunomodulatory effects. Nevertheless, more research is expected to unveil novel functional components and mechanisms and validate these beneficial effects. Enhanced biosafety and technological and economic advantages make mineral-enriched postbiotics ideal candidates as adjuvant therapy, preventing the origin of disease condition or ameliorating symptoms. 

## Figures and Tables

**Table 1:** Beneficial effects of postbiotics (inactivated cells, cell fractions, or metabolites).

Model	Effect	Components	Reference
Clinical study	Potentially reduced abdominal pain and modification of stool consistency for patients with irritable bowel syndrome	Non-viable probiotic lysate of *Escherichia coli* DSM 17252 and *Enterococcus faecalis* DSM 16440	[15]
In vitro	Inhibition of lipid accumulation during adipocyte differentiation	Cell lysate of *Lactobacillus plantarum* K8	[16]
In vitro	Antibacterial and antioxidant activity	The cell-free supernatant from *L. plantarum* RG11, RG14, RI11, RS5, TL1, and UL4	[17]
In vitro	Antibacterial and antibiofilm activity	The cell-free supernatant from *L. sakei* EIR/CM-1	[18]
In vitro	Antimicrobial activity	Metabolic products of *L. acidophilus* LA5, *L. casei* 431, and *L. salivarius*	[19]
In vitro	Increased interleukin IL10 concentration and expression CD103 and CD1d Downregulated expression of NFκB1, RELB, and TNF genes Influenced retinoic acid—driven mucosal-like dendritic cells	The cell-free supernatant from *L. reuteri* DSM 17938	[20]
In vitro and animal model mice	Immunomodulatory effect	Milk fermentation product using *Brevibacterium breve* C50 and *Streptococcus thermophiles* 065	[21]
Animal—mice	Improving the parameters associated with intestinal mucositis induced by chemotherapy	Heat inactivated cells of *L. rhamnosus*	[22]
Animal—rat	Preventing periodontitis by reducing alveolar bone loss and ameliorating the bone microarchitecture parameters	Heat inactivated cells of *L. reuteri*	[23]
Animal—broiler chickens	Immunomodulatory effect on jejunal tissue Decreased *Clostridium perfringens* colony counts, decreased lesions scores, and mortality	Fermented product produced from a consortium containing *Pediococcus acidilactici*, *L*. *reuter*, *Enterococcus faecium*, and *L. acidophilus*	[24]
Animal—suckling rat	Protection against rotavirus infection	Cell fragments and metabolites of probiotic microorganisms and prebiotics	[25]
Animal—post-weaning lambs	Immunomodulatory effect (increase of IL-6, decrease of IL1 and TNF) Pathogenic bacteria inhibition	The cell-free supernatant from *L. plantarum* RG14	[26]
Animal—broiler chickens	Immunomodulatory effect Reduce cell number of *Enterobacteria* and *E. coli*	The cell-free supernatant from *L. plantarum* RG14 probiotic microorganisms and inulin	[27,28]
Animal—broiler chickens	Improving growth performance Reduced number of *Enterobacteriaceae* Increase expression of hepatic IGF-1 and GHR mRNA and plasma immunoglobulins (IgG and IgM)	The cell-free supernatant from *L. plantarum* RI11, *L. plantarum* RS5 and *L. plantarum* UL4	[29,30]
Animal—neonatale rat	Promoting mucin secretion and the epithelial tight junction protein expression	Components of the cell-free supernatant from *L. rhamnosus* GG	[31]
Animal—rat	Increase the global bone mineral density	Bacterial lysate and supernatant from *L. acidophilus, L. casei, L. reuteri*, *Bifidobacterium longum*, and *Bacillus coagulans*	[32]

## Data Availability

Graphical Abstract and Figure 1 and Figure 2 were created with BioRender.com (accessed on 24 August 2022).
